# Managing health systems under armed conflict: a scoping review of international evidence and a document review of Iranian experiences

**DOI:** 10.1080/16549716.2026.2717482

**Published:** 2026-08-18

**Authors:** Neda Kabiri, Mina Mahami-Oskouei, Hanieh Salehi-Pourmehr, Kavous Shahsavari Nia, Sakineh Hajebrahimi

**Affiliations:** a Research Center for Evidence-Based Medicine, Faculty of Medicine, Tabriz University of Medical Sciences, Tabriz, Iran; b Department of Medical Library and Information Science, School of Management and Medical Informatics, Tabriz University of Medical Sciences, Tabriz, Iran; c Emergency Medicine Research Team, Tabriz University of Medical Sciences, Tabriz, Iran

**Keywords:** Armed conflict, wartime, health management, financial management, digital health

## Abstract

Wars and armed conflicts are among the most critical crises that can severely impact a country’s vital infrastructures, particularly the health system. During wartime, health systems face major challenges, including attacks on healthcare facilities and personnel, which disrupt access to services and undermine public health. This study aims to conduct a scoping review to assess reported international strategies for wartime health system management, focusing on resource allocation, workforce distribution, financing, and access to essential supplies, followed by a document review of Iranian experiences to inform policy recommendations. We used the JBI framework for the scoping review phase. Relevant English and Persian studies were systematically searched across multiple databases without time limitation, and data were analyzed using qualitative content analysis. We included 26 international studies and 27 Iranian documents in our study. Findings showed that health systems in armed conflict settings face multiple challenges, including damaged infrastructure, shortages of health workforce, disruptions in essential services, limited financial resources, psychological stress on staff, and reduced patient access to care. Interventions such as digital health tools, e-learning, intersectoral collaboration, national and international networks, and community-based approaches were commonly reported as strategies that may support service continuity and resilience in conflict settings. Key recommendations for building resilient health systems in conflict settings include systematic emergency preparedness and coordination plans, decentralization of essential health services to ensure continuity of care, strategic investment in workforce capacity and mental health support, and the establishment of mechanisms that foster flexibility, learning, and international collaboration.

## Paper Context

**Main findings:** Armed conflicts severely disrupt health systems through infrastructure damage and workforce shortages, yet evidence shows that digital health innovations, decentralized service delivery, and community-engaged responses can sustain essential care during wartime.

**Added knowledge:** By integrating international experiences with national documents, this study provides a practical framework for adapting proven resilience strategies to the unique political, cultural, and resource contexts of countries at risk of armed conflict.

**Global health impact for policy and action:** These findings equip health leaders and policymakers with actionable priorities such as pre-crisis coordination plans, flexible financing mechanisms, and cross-border knowledge sharing, to safeguard health systems and protect vulnerable populations in an era of increasing geopolitical instability.

## Background

War and armed conflict are among the most severe crises capable of profoundly disrupting a country’s critical infrastructure, particularly its health system. In situations of armed conflict, health systems face acute and multidimensional challenges: attacks on healthcare facilities and health personnel not only interrupt access to essential services but also may undermine population health and system performance [1]. In such contexts, the demand for healthcare services typically rises sharply, while infrastructure, medical supplies, financial resources, and trained personnel become increasingly constrained. Experiences from conflict-affected countries demonstrate that, in the absence of adequate preparedness, health systems may rapidly deteriorate, placing the lives of civilians, patients, and healthcare workers at serious risk [2].

In the contemporary global context, several countries have shown that through the design of appropriate institutional mechanisms and deliberate strengthening of health system resilience, it is possible to sustain essential services even during wartime. Reviewing these experiences may assist countries that are not currently engaged in armed conflict but face potential threats in developing preventive strategies and preparedness frameworks [3]. Health system resilience in wartime refers to the capacity to maintain, adapt, and restore critical functions in the face of violence, infrastructure destruction, and resource scarcity. A resilient health system must not only ensure the continued provision of essential services but also rapidly adjust to evolving wartime dynamics, including population displacement and targeted attacks on healthcare facilities. Strengthening resilience therefore requires anticipatory planning, workforce training, mobilization of local capacities, and the establishment of flexible service delivery pathways. Evidence from multiple settings suggest that health systems characterized by adaptive structures, strong leadership, and effective community engagement may be better positioned to withstand conflict-related shocks and save lives [4,5].

A study conducted in Ukraine demonstrated that coordinated governmental efforts to preserve public financing for primary healthcare were associated with the prevention of systemic collapse during wartime. The study further highlighted how strategic actions undertaken during the COVID-19 pandemic, such as the expansion of digital communication platforms and the strengthening of workforce competencies, were reported as contributing factors to resilience during the subsequent armed conflict. The authors emphasized the importance of managerial and financial autonomy in enabling rapid and effective organizational responses to crisis. At the same time, emerging challenges were identified, including ensuring equitable access to primary healthcare among internally displaced persons, shifting patient profiles and service needs, and vulnerabilities associated with reliance on local government financing. Ultimately, the study recommended coordinated planning and sustained development-oriented efforts to secure the long-term effectiveness of primary healthcare services in Ukraine [6].

Another study, based on in-depth interviews with Syrian health actors operating from Turkey into northwest Syria, examined the functioning of cross-border humanitarian health operations. The findings suggested that transferring responsibility for healthcare delivery from international non-governmental organizations to local actors in conflict settings may improve operational efficiency when supported by bottom-up strategies, social trust, and high levels of contextual risk tolerance. However, the absence of formal documentation, systematic evaluation, and adequate protection for local health workers was identified as a significant structural challenge [7]. A further study in Syria emphasized the need for participatory approaches that empower local stakeholders and civil society organizations, enhance collaboration between national and international actors, improve access to data, and establish a central coordinating body for planning and oversight during wartime. The authors concluded that investment in data systems, mobile health technologies, e-health solutions, capacity building, and strong independent technical leadership may contribute to strengthening humanitarian health responses in Syria and other crisis-affected regions [8].

Given its geopolitical position and historical experience of the Iran–Iraq war, as well as more recent regional hostilities, Iran faces a pressing need to reassess its health system preparedness for crisis conditions. Nevertheless, comprehensive and applied studies that integrate global experiences with the specific institutional and contextual realities of Iran remain limited. This study therefore seeks to bridge this gap by systematically examining published international experiences in wartime health system management, such as, resource allocation, workforce distribution, financing of equipment, and access to medicines and essential medical supplies, and subsequently conducting a document review of national experiences. The ultimate aim of this study is to propose a set of practical, evidence-informed, and contextually adaptable policy recommendations to enhance the resilience and preparedness of Iran’s health system in the face of potential armed conflict.

## Methods

This study consisted of two phases: a scoping review of international evidence and a document review of Iranian experiences.

### Phase 1: scoping review of international evidence

In the first phase, a scoping review was conducted to identify and synthesize international evidence on health system management during war and armed conflict. The scoping review was conducted according to the JBI (previously Joanna Briggs Institute) instructions [9]. Reporting of this review was guided by the Preferred Reporting Items for Systematic Reviews and Meta-Analyses extension for Scoping Reviews (PRISMA-ScR) checklist [10].

#### Inclusion and exclusion criteria

Based on the PCC framework (Population, Concept, and Context) used in scoping reviews, the inclusion and exclusion criteria were defined as follows:

- P (Population): In this study, the population included health systems, healthcare organizations, healthcare providers, policymakers, and populations affected by war or armed conflict.
- C (Concept): The concept focused on health system management during war and armed conflict, including resource allocation, human resource distribution, financial support for equipment, and access to essential medicines and medical supplies. It also includes strategies, programs, and policies adopted by these countries to manage their health systems during crisis conditions.
- C (Context): The context includes all countries or regions affected by war, armed conflict, or conflict-related humanitarian crises.

#### Type of studies

Original qualitative, quantitative, and mixed-methods studies, systematic and scoping reviews, case studies, and policy papers were included. Editorials, conference abstracts, letters, and commentaries without relevant empirical or policy evidence, as well as publications unrelated to health system management in conflict settings, were excluded. No time and language restrictions were applied. Abstracts of publications in languages other than English were translated using Google Translate before screening and data extraction.

#### Search strategy

Following the identification of search keywords based on the study question, both controlled and free keywords including ‘health system resilience, war, violence, and conflict’ were used to perform structured searches across the following databases: MEDLINE (via Ovid), Embase, PubMed, CINAHL (via EBSCO), Scopus, and ISI Web of Science. The search was conducted in September 2025. The full search strategy for PubMed is as below:

((((((Armed Conflicts[MeSH Terms]) OR (Armed Conflict*[Title/Abstract])) OR (Wars[Title/Abstract])) OR (Atrocities[Title/Abstract])) OR (terrorist threats[Title/Abstract])) OR (wartime[Title/Abstract])) AND ((((((((((health system resilience[Title/Abstract]) OR (health system management[Title/Abstract])) OR (human resource*[Title/Abstract])) OR (Staffing*[Title/Abstract])) OR (financial management[Title/Abstract])) OR (Financial Activit*[Title/Abstract])) OR (Workforce*[Title/Abstract])) OR (Workforce[MeSH Terms])) OR (Financial Management[MeSH Terms]) OR resource generation[Title/Abstract] OR stewardship[Title/Abstract] OR healthcare provision[Title/Abstract]))). The full search strategy for Scopus, ISI Web of Science, and Embase is provided in the Additional file 1.

#### Study selection

All identified studies were imported into EndNote 21, and duplicates were removed. The selection process was carried out based on the inclusion and exclusion criteria through three stages: title, abstract, and full-text screening. All titles and abstracts were independently reviewed by two reviewers. Potentially relevant articles were retrieved in full for detailed examination. If additional information was required for decision-making, the authors of the selected studies were contacted. Any disagreement between reviewers at any stage was resolved through discussion.

#### Data extraction

Data extraction was performed independently by two reviewers using a Microsoft Excel data extraction form developed for this review. As recommended by the JBI scoping review methodology, descriptive information about the sources, such as author(s), citation, title, year of publication, study objective, study type, country, and setting, was recorded. Main data extracted included types of policies or programs implemented for health system management and any other key information relevant to the research questions and inclusion criteria.

#### Data synthesis

Data synthesis was conducted using a conventional qualitative content analysis approach [11]. Codes were generated inductively from the extracted findings of the included studies. Similar codes were grouped into subcategories and then synthesized into broader categories. The initial coding was performed by the first author and subsequently reviewed by the research team. Any disagreements regarding the coding or categorization were resolved through discussion until consensus was achieved.

### Phase 2: document review of Iranian experiences

In the second phase of this study, a document review was conducted to identify and synthesize national experiences related to health system management during Iranian wars, including the eight-year Iran–Iraq War, which began in September 1980, and the 12-day war between Iran and Israel, which lasted from 13 to 24 June 2025.

A search was performed in Persian using combinations of keywords related to war, armed conflict, health system, healthcare, the Ministry of Health, hospitals, crisis management, emergency care, health workforce, medicines and medical supplies, and resilience. Official reports, policy documents, statements, interviews with health authorities and experts, news reports containing official interviews, and other publicly available documents published by governmental organizations, professional associations, and universities were reviewed. Searches were conducted through the Google search engine and the official websites of the Iranian Ministry of Health and Medical Education, medical sciences universities, the Health Commission of the Islamic Consultative Assembly (Majlis), the Iranian Medical Council, the Supreme Council of the Iranian Nursing Organization, hospital emergency departments, emergency supervisors, the Passive Defence Committee, the Majlis Research Center, the Iran Health Insurance Organization, and the State Welfare Organization.

Documents were eligible if they described experiences, challenges, management strategies, response measures, lessons learned, or policy recommendations related to health system management during wartime or armed conflict in Iran. Documents that lacked sufficient information regarding health system management or represented duplicate reports of the same event were excluded.

All eligible documents were screened by title and full text. Relevant information was extracted using a standardized data extraction form that included the source, author(s), conflict context, reported challenges, health system strengths, and policy recommendations. The extracted data were analyzed using conventional qualitative content analysis. Similar concepts were grouped into categories and themes through an iterative coding process, and the findings were synthesized narratively.

## Results

### Study inclusion

#### Phase 1: scoping review of international evidence

The database search yielded a total of 1926 records. After removal of 350 duplicate records, 1576 titles and abstracts were screened for eligibility. Of these, 1482 records were excluded based on inclusion and exclusion criteria. Subsequently, 94 full-text articles were assessed for eligibility, resulting in the inclusion of 26 studies in the final analysis. Studies were excluded due to irrelevance to the study objectives, insufficient data for extraction, or lack of focus on health system management during armed conflict.

#### Phase 2: document review of Iranian experience

**Figure 1.**
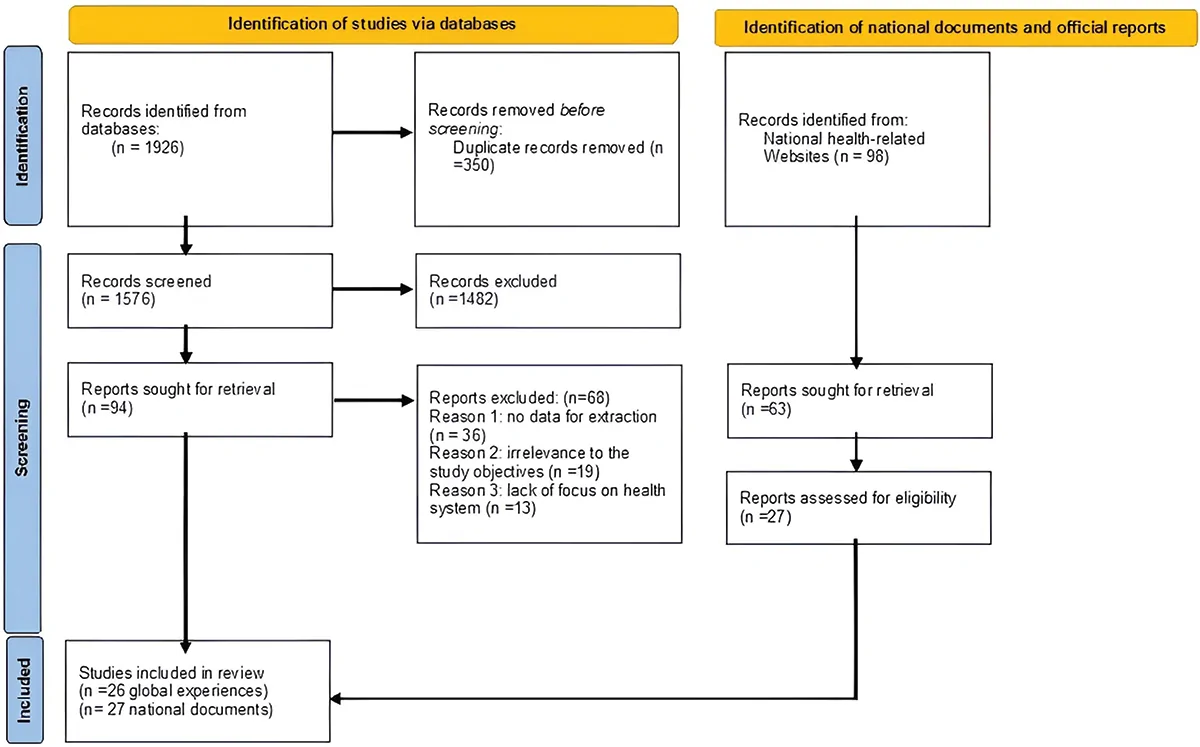
PRISMA flow diagram.

The search for Iranian national documents identified 98 potentially relevant records from official websites, governmental reports, and organizational documents. After screening titles and full texts and removing irrelevant or duplicate records, 27 documents met the eligibility criteria and were included in the document review. These documents represented official statements, reports, interviews, and policy documents describing the experiences of the Iranian health system during armed conflicts. The study selection process for the scoping review and document review is summarized in Figure 1.

#### Main findings of phase 1: scoping review of international evidence

##### Main characteristics of included studies

In the scoping review phase, which aimed to identify reported experiences in health system management during crisis and armed conflicts, studies were included from Ukraine [6,12–16], Sudan [17–19], Gaza [20,21], Yemen [22,23], Nepal [24], Myanmar [25], Cote d’Ivoire [26], Nigeria [27], Rwanda [28], and Uganda [29]. Also, seven multi-country studies examined cross-national experiences [2,30–35].

Main characteristics of included studies in the scoping review are presented in Additional file 2.

##### Challenges of healthcare system in conflict settings

The qualitative content analysis of the included studies in the scoping review phase revealed that health systems in conflict settings face multidimensional, interrelated, and systemic challenges. Ukraine primarily faced repeated attacks on health facilities, disruption of healthcare services, shortages of health professionals, increasing mental health needs, changing patterns of disease, and barriers to healthcare access among displaced populations [6,12–16]. Sudan experienced one of the most extensive system-wide disruptions, characterized by health system collapse, workforce shortages, service interruptions, destruction of medical education, financial constraints, and inadequate international support [17–19]. In Gaza, the most critical challenges included severe shortages of essential medicines, medical equipment, and trained personnel, along with overwhelming patient surges following military attacks [20,21]. Yemen was mainly challenged by insecurity, restricted access to health services, politicization of humanitarian assistance, weak health system capacity, and cholera outbreaks [22,23]. Other conflict-affected settings reported additional challenges, including damaged health infrastructure and loss of qualified health workers in Côte d’Ivoire [26], communicable disease outbreaks and shortages of specialists in post-genocide Rwanda [28], vaccine delivery barriers and low uptake of vaccine among the population due to fear of conflict in rural Nepal [24], and disruption of maternal and child health services across multiple conflict-affected countries (Afghanistan, Colombia, Congo, Mali, Nigeria, Pakistan, Somalia, South Sudan, Syria, Yemen) [33]. Global evidence indicated common systemic vulnerabilities, including inadequate emergency preparedness, weak governance, insufficient financing, workforce migration, limited health information systems, and poor digital infrastructure, all of which have been reported to undermine health system resilience during armed conflicts [2,30–35].

These challenges were categorized into eight major domains of destruction and disruption of health infrastructure and services, human resources crisis, disruption of medical education, shortage of medicines, equipment, and essential supplies, governance, preparedness, and systemic weaknesses, epidemiological shifts and increased disease burden, disruption of vaccination and public health programs, and digital and health information system barriers. These categories and related subcategories are displayed in Table 1.

**Table 1.** Main categories and subcategories of health system challenges during war (results of the scoping review phase).

Main category	Subcategories
Destruction and disruption of health infrastructure and services	Military attacks on health facilities (2254 attacks); 60.7% facilities inactive; 46.2% structures damaged; looting and military use of buildings; disruption of services; interruption of care; surge of patients after missile attacks; direct risk to emergency staff; reduced access to services; pressure on PHC; limited access for displaced populations [2,14,20,34]
Human resources crisis	Reduction of health workforce; 77.7% qualified staff left; migration due to insecurity; shortage of trauma, prosthetics and rehabilitation specialists; psychological pressure; physical and mental impact on NGO staff; inadequate payments; shortage of training; disrupted supervision; limited educational capacity [12,26,31,32]
Disruption of medical education	Destruction of medical education; disruption of classes, laboratories and clinical training; lack of educational resources; reduced morale among students and faculty; limited educational capacity [25,29,35]
Shortage of medicines, equipment, and essential supplies	Severe shortages of essential medicines (insulin, anti-epileptics, cancer drugs, antibiotics, psychotropics); lack of oxygen and diagnostic tools; financial shortages; dependence on unstable local funding; high service costs; inadequate electricity supply; shortage of computers and internet modems; costly access to digital tools [13,16,20,35]
Governance, preparedness, and systemic weaknesses	Lack of coherent governance; weak resilience; insufficient preparedness for sudden shocks; weak early warning systems; absence of minimum emergency response plans; inadequate international response; politicization of aid; mistrust; security and political barriers; limited system capacity; poor-quality data; limited reporting [2,16,23,30,34]
Epidemiological shifts and increased disease burden	Cholera outbreaks; increase in measles and STIs; emergence of gastritis and trauma-related conditions; HIV, malaria, TB spread; increased mental health needs (46% affected); rise in mental and neurological disorders (41%, 39%); NCDs accounting for 84% of deaths; changing disease profile (greater NCD and psychosocial needs); disruption of maternal and child services; lack of stillbirth monitoring; inadequate adolescent care [16,24,28,33]
Disruption of vaccination and public health programs	Risk during vaccine transport; reduced vaccination uptake due to fear; incomplete maternal and child health services [16,24,34]
Digital and health information system barriers	Weak and unreliable internet; challenges implementing health IT; lack of confidence and skills in IT use; insufficient computers; electricity shortages; limited and costly digital access [13,18,29,35]

##### Strategies and interventions for health system resilience in conflict settings

The reviewed studies identified a wide range of strategies reported by conflict-affected countries to maintain health system functioning during war. Ukraine emphasized strategies such as strengthening individual resilience and system flexibility [12], expanding digital health solutions to maintain continuity of care for vulnerable populations [13], utilizing lessons learned from the COVID-19 pandemic [6], strengthening non-governmental organizations (NGOs) with adequate resources [15], and increasing managerial and financial autonomy to enable rapid response [6]. Sudan implemented comprehensive interventions including strengthening supply chains, decentralizing resources, promoting intersectoral collaboration, expanding community-based approaches, crisis-sensitive financing, and workforce capacity building and training [17]. Sudan also adopted innovative educational strategies, such as online learning, small group learning, mobile clinical learning, collaboration among universities, overseas training, international academic partnerships, and regional training centers to sustain medical education during conflict [18,19]. In Gaza, mobile medical service points were reported as an approach to improve access to essential healthcare in disrupted settings [20]. Yemen reported efforts focused on monitoring service quality, optimizing staffing and organizational structures, improving service coordination, and strengthening human resource development and supply management [22,23]. Rwanda prioritized community-based healthcare, decentralization of services, accountable workforce development, and investment in health information technologies [28]. Nigeria concentrated on maintaining essential public health and clinical services, including immunization, integrated management of childhood illnesses focusing on malaria, pneumonia, malnutrition, diarrhea, maternal and newborn care, reproductive health, non-communicable disease management, and mental health services [27]. Across multiple conflict-affected countries, maintaining essential maternal and child health services and adopting innovative humanitarian approaches to bring healthcare closer to affected populations were frequently reported priorities [33]. Global evidence further highlighted reported strategies for strengthening emergency preparedness, enhancing health system absorptive, adaptive, and transformative capacities, improving stakeholder coordination, reinforcing health information systems, engaging communities, and establishing sustainable financial and governance mechanisms to support health system resilience during armed conflict [2,30–35].

These identified interventions for health system resilience in conflict settings were categorized into nine major domains: workforce strengthening, digital health expansion, service continuity prioritization, pharmaceutical and supply chain stabilization, governance reform and decentralization, emergency preparedness, adaptation of health professional education, community engagement, and post-conflict reconstruction (Table 2).

**Table 2.** Strategies and interventions identified in international experiences of health system management during conflict (scoping review phase).

Main category	Subcategories
Health workforce strengthening and protection	Capacity building and workforce training; Emergency skills training; Retention and return of qualified staff; Incentive policies for health workers; Community health workforce engagement; Local workforce utilization; Psychological support for frontline providers; Development of context-sensitive HR policies; Reinforcement of NGOs with adequate resources [12,15,17,23,24,26,28,31,32]
Digital health and e-learning innovations	Deployment of digital health platforms (e.g. ARTporuch#); Telemedicine expansion; E-learning for medical education; Remote and hybrid education models (PBL, TBL, small groups); Health information systems strengthening; Investment in health IT infrastructure; Digital communication tools during crisis [6,13,18,29,35]
Service continuity and essential health package prioritization	Prioritization of PHC services; Maintenance of HIV, NCD, maternal and child health services; Vaccination programs; IMCI implementation; Emergency obstetric and neonatal care (EmONC/BEmONC); Mental health and psychosocial support; Adaptive service delivery models; Community-based service provision; Establishment of mobile medical points [16,20,27,28,33]
Supply chain and pharmaceutical system strengthening	Strengthening supply chains; Decentralization of medicine distribution; Crisis-sensitive financing; Investment in supply management systems; Ensuring access to essential medicines; Removal of financial barriers to services [16,17,23]
Governance, coordination, and decentralization	Decentralization of health services; Strengthening governance and stewardship; Intersectoral collaboration; Stakeholder coordination; Integrated humanitarian-development approach; Participatory and community-engaged governance; PDCA continuous improvement cycle; Transformative resilience approaches (absorptive, adaptive, transformative capacities) [2,14,25,28,30,34]
Emergency preparedness and system resilience	Development of minimum emergency response plans; Integration of preparedness into health policy; Surge capacity enhancement; Organizational flexibility; Risk reduction policies; Early integration of recovery planning; Crisis-adapted financing mechanisms [2,14,30,34]
Education system adaptation in conflict settings	Online education models; Regional education hubs; International academic collaboration; Protection of students and faculty; Reconstruction planning for academic infrastructure [18,19]
Community engagement and humanitarian collaboration	Community participation mechanisms; Empowerment of local actors; NGO strengthening; Citizen and civil society involvement; Trust-building initiatives; Alignment of humanitarian and development actors [14,23,28,31,33]
Recovery and post-conflict health system reconstruction	Reconstruction of damaged facilities; Return of qualified staff; Integration of resilience into rebuilding; Equity and gender-responsive recovery; Climate and disaster resilience integration; Alignment with national reconstruction frameworks [26,28,34]

#### Main findings of phase 2: document review of Iranian experiences

##### Acute wartime challenges and long-standing structural weaknesses of the Iranian healthcare system

The review of Iranian documents showed that the health system faced both acute wartime challenges and pre-existing structural weaknesses. Immediate challenges included the sudden onset of the conflict, large numbers of casualties within a short period, direct attacks on hospitals and healthcare facilities, logistical difficulties, temporary disruptions in pharmaceutical supply chains, communication outages, and substantial physical and psychological pressure on healthcare workers. Several reports also highlighted operational challenges such as the absence of protected hospitals and shelters, transportation difficulties for healthcare personnel, and the need to prepare for chemical and radiological incidents. On the other hand, the documents identified long-standing structural weaknesses within the Iranian healthcare system, including shortages and unequal distribution of human resources, aging hospital infrastructure, weak emergency preparedness, fragmented crisis governance and the absence of a unified command structure, limited public preparedness and health literacy, insufficient organization of volunteers, financial constraints, and inadequate legal and organizational mechanisms for community participation. Vulnerable populations, particularly older adults, also faced barriers related to disrupted access routes, shortages of medicines and essential supplies, and limited availability of specialized services. These findings suggest that while the conflict exposed important operational challenges, many of the identified weaknesses were related to underlying systemic limitations that predated wartime conditions and became more apparent during crises. Main characteristics of included national documents and official reports are presented in Additional File 3.

The main challenges and weaknesses are summarized in Table 3.

**Table 3.** Challenges and weaknesses of the Iranian health system during war (document review phase).

Category	Key challenges
Sudden shock and surge capacity limitations	Unexpected onset of war; Simultaneous multi-site attacks; Overwhelming casualty volume; Capacity mismatch in peak operations (e.g. Karbala-4); Heavy hospitalization pressure; Continuous admission burden
Structural vulnerability of hospitals	Direct attacks on hospitals and ambulances; Damage to seven hospitals; Evacuation of facilities; Over 50% hospital structural aging; One-third requiring reconstruction; Lack of underground/safe hospitals; Lack of protected shelters; Absence of comprehensive secure centers in major cities
Workforce burnout and structural imbalance	Chronic nursing shortages; Unequal geographic distribution; Severe psychological stress (especially nurses); Physical and mental fatigue; Threat to staff safety; Concerns about families; Delayed payments; Inequitable payment systems; Ignored post-crisis demands
Governance and command fragmentation	Lack of unified crisis command; Weak participatory management structure; Slow operational response; Resource fragmentation; Weak legal mechanisms for public participation; Poor volunteer organization in early phase; Structural weakness in some associations
Logistics and supply vulnerabilities	Initial disruption in drug and blood supply; Raw material shortages; Price conflicts between producers and authorities; Transportation challenges; Staff accommodation issues; Risk of shortages in prolonged crisis; Limited shortages in specific drugs (e.g. chemotherapy); Access disruption for elderly populations
Infrastructure and utility risks	Internet outages; Communication restrictions; Risk of electricity, water, gas disruption; Dependence on electronic systems
Financial and economic pressure	Budget constraints; Financial deductions; War and sanctions pressure; Increased public demand for services
Preparedness gaps for modern warfare	Limited preparedness for chemical/radiological attacks; Lack of codified military medicine doctrine; Initial inexperience in chemical exposure management; Distance between frontline and treatment centers in early war phase; Insufficient public crisis education; Lack of formal planning for maintaining PHC centers during crisis
Community-level and behavioral challenges	Public gathering at incident sites; Low crisis health literacy; Population shifts to host provinces; Increased vulnerability of elderly; Political sensitivity and media self-censorship

##### Health system strengths and resilience capacities in Iran

The review of Iranian documents demonstrated several major resilience capacities that supported the health system’s ability to maintain service delivery during wartime. Across almost all documents, the most consistently reported strength was the exceptional commitment, solidarity, and continuous presence of healthcare workers, including physicians, nurses, emergency personnel, students, retirees, and volunteers, who maintained services despite heavy workloads, security threats, and personal hardships. Health services remained operational throughout the conflict, with hospitals sustaining inpatient and outpatient care, performing large numbers of emergency surgeries, and managing thousands of casualties with limited reported interruptions. Rapid activation of emergency preparedness and incident command mechanisms were another major strength of the health system. National and provincial crisis headquarters were established immediately, emergency protocols and mass casualty management guidelines were disseminated within hours, trauma teams and crisis committees were activated, hospitals expanded surge capacity, and extensive staff training was rapidly implemented through webinars, drills, and operational guidelines. The documents also highlighted strong intersectoral coordination among the Ministry of Health, medical universities, the Food and Drug Organization, pharmaceutical manufacturers, relief agencies, the armed forces, insurers, and other governmental organizations. This coordinated governance supported rapid decision-making, efficient allocation of resources, nationwide redistribution of medical supplies, and effective communication between central and local authorities. Another important resilience capacity was the robustness of pharmaceutical and logistics management. Despite concerns regarding sanctions and wartime disruption, coordinated supply chain management, substantial domestic pharmaceutical production, rapid collaboration with manufacturers and distributors, and continuous monitoring of inventories were reported to help prevent major shortages of essential medicines, medical equipment, blood products, and infant formula during the conflict. The document review also demonstrated considerable flexibility in service delivery. Hospitals rapidly expanded treatment capacity, mobilized additional personnel from other provinces, established field hospitals and mobile medical units, utilized military hospital capacity when necessary, and adapted clinical pathways to maintain continuity of essential services while responding to mass casualty incidents. Finally, previous experience gained during the Iran–Iraq War, the COVID-19 pandemic, and repeated national emergency preparedness exercises was reported as contributing to institutional readiness. Existing operational knowledge, trained emergency response teams, established volunteer networks, digital coordination systems, community participation, and accumulated crisis management experience supported rapid respond and continuity of essential health services during the conflict. These findings were synthesized into eight major categories of health system resilience capacities identified from the Iranian document review, as presented in Table 4.

**Table 4.** Major resilience capacities and strengths of the Iranian health system during wartime (document review phase).

Category	Key findings
Health workforce capacity and commitment	Continuous presence of healthcare workers at all levels; voluntary participation; high professional commitment and self-sacrifice; participation of private-sector physicians; mobilization of retired staff and students; experience from Iran-Iraq war and COVID-19 pandemic
Continuity of essential health services	Uninterrupted inpatient and outpatient services; management of over 5,000–8,000 injured patients; 1,064 surgeries within 12 days of Iran-Israel war; maintenance of elective services in some centers; free care for war casualties; continued operation of 29,000 diagnostic and treatment centers
Emergency preparedness and operational response	Immediate activation of emergency protocols; 100% emergency readiness; implementation of Mass Casualty Management (MCM); hospital incident command systems; crisis committees; trauma teams; standby hospitals; optimized triage; rapid patient evacuation; reduced casualty transfer time; deployment of field hospitals and frontline medical teams.
Pharmaceutical and supply chain resilience	Rapid activation of the Food and Drug Crisis Headquarters; uninterrupted supply of medicines, medical equipment, and infant formula; coordinated procurement and distribution; strong domestic pharmaceutical production; emergency stockpiling; rapid restoration of disrupted production and distribution systems.
Leadership, governance, and intersectoral coordination	Centralized crisis command; strong leadership by the Ministry of Health; coordination among medical universities, governmental organizations, armed forces, military hospitals, insurers, and humanitarian partners; organized volunteer management; efficient redistribution of financial and operational resources; documentation of attacks on healthcare facilities.
Infrastructure and operational logistics	Preventive logistics planning; emergency power systems; strategic stockpiling of drugs and food; allocation of backup hospital capacity; rapid replacement of damaged facilities; continuous operational readiness.
Community engagement and social solidarity	Broad mobilization of volunteers and civil society; neighborhood support networks; collaboration between public and private sectors; community-based support for vulnerable populations; educational materials for stress management; strong social cohesion during the crisis.
Health information and communication coordination	Internal communication and coordination mechanisms; public health information dissemination; crisis reporting and documentation; digital support for coordination and decision-making.

## Discussion

The findings of this study indicate that health system resilience in wartime is not the product of a single intervention, but rather the outcome of a dynamic interaction among workforce policies, governance arrangements, financing mechanisms, infrastructure, technology, and community engagement. International evidence suggests that health systems are better able to withstand severe shocks when they simultaneously cultivate the capacities to absorb, adapt, and transform [36–38]. A resilient health system requires not only the ability to maintain essential functions during acute crises but also the capacity to reorganize and strengthen structures to address future threats.

### Health workforce strengthening and protection

Within this conceptual framework, strengthening the health workforce emerges as the foundational pillar of any resilience strategy. Even where infrastructure and material resources are available, the absence of adequately trained, motivated, and supported personnel can paralyze service delivery. Research conducted in fragile and conflict-affected settings shows that retention policies, financial and non-financial incentives, emergency skills training, and psychosocial support for frontline staff may contribute to workforce retention and better patient outcomes [39–41]. However, workforce sustainability cannot be achieved in isolation; coherent governance structures and supportive institutional environments are essential. Evidence suggests that decentralization of services and meaningful engagement of local stakeholders in decision-making processes may enhance operational flexibility and response capacity [42].

Our findings regarding workforce-related challenges are consistent with previous evidence from Iran. A recent scoping review on health workforce challenges in the Iranian healthcare system identified persistent structural problems, including shortages and unequal distribution of health professionals, heavy workload, burnout, inadequate financial incentives, limited workforce retention, and weaknesses in human resource planning and management. These pre-existing challenges may become more pronounced during armed conflict, when health systems face increased casualty loads, direct security threats, and prolonged operational demands. Similarly, our review of both international experiences and Iranian documents demonstrated that workforce shortages, psychological distress, physical exhaustion, and concerns about staff safety were among the most frequently reported challenges [43].

### Continuity of essential health services and protection of vulnerable populations

In this context, continuity of essential services, particularly primary care, HIV services, non-communicable disease management, and maternal and child health, constitutes the backbone of health system survival. Empirical studies indicate that disruptions to these core services are associated with indirect mortality that may exceed direct conflict-related deaths [44,45]. Similar to experiences during the COVID-19 pandemic, our findings indicate that large-scale crises require health systems to rapidly reorganize services, maintain continuity of essential care, and strengthen resilience through adaptive management and coordinated responses [46]. Therefore, prioritizing an essential service package and implementing flexible delivery models, such as mobile clinics and community-based platforms, may support not only the maintenance of access but also the protection of health equity. Achieving this continuity depends fundamentally on resilient supply chains and pharmaceutical systems. The literature suggests that decentralized drug distribution, strategic stockpiling, and strengthening domestic production capacity may reduce the risk of shortages of essential medicines in crisis contexts [47]. At the same time, crisis-sensitive financing mechanisms and the removal of financial access barriers have been reported to facilitate service utilization among vulnerable populations [48]. Our findings indicated that in low-income countries such as Iran, where health systems often face shortages of financial resources, infrastructure, and health workers even before conflict, maintaining essential health services may rely on community participation, humanitarian support, task shifting, and strengthening primary health care. However, in middle-income countries, such as Ukraine, existing health infrastructure, domestic pharmaceutical production, previous disaster experience, and established referral systems have been reported as factors that may support a more coordinated emergency response and faster recovery.

Another important finding of this review is that health system resilience during armed conflict should be viewed not only as the ability to maintain services but also as the capacity to ensure equitable access to care for vulnerable populations. Across the included studies in the scoping review phase, internally displaced persons, women, children, older adults, people living with HIV, patients with chronic diseases, and individuals requiring mental health services were disproportionately affected by disruptions in health service delivery. Several studies reported reduced access to primary care, maternal and child health services, essential medicines, rehabilitation, and long-term treatment for non-communicable diseases. Innovative strategies such as decentralized service delivery, mobile medical units, digital health platforms, community-based approaches, and prioritization of essential health services were frequently reported as approaches to improve access for vulnerable populations. The Iranian experience similarly emphasized maintaining free access to emergency care, uninterrupted pharmaceutical supply, nationwide service continuity, and coordinated referral systems during the conflict. These findings suggest that future wartime preparedness plans may benefit from explicitly incorporating equity considerations by prioritizing the needs of vulnerable populations, ensuring continuity of essential services, strengthening community-based approaches, and reducing geographical, financial, and organizational barriers to care.

### Governance, leadership, and emergency preparedness

Our findings from the Iranian experience suggest that several challenges observed during the conflicts were not solely war-related but reflected pre-existing and long-lasting structural weaknesses within the health system. Similarly, a systematic review of leadership and governance policies in Iran identified structural shortcomings, including fragmented governance, inadequate coordination, limited stakeholder engagement, and weaknesses in policy implementation, all of which may undermine health system resilience during emergencies. These findings indicate that strengthening governance and institutional capacity should be considered as a long-term resilience strategy, rather than a response implemented only during crises [49].

### Digital health and health information systems

Digital health plays an integrative role within this ecosystem, functioning as a connective infrastructure across system components. Telemedicine can bridge specialist gaps in conflict-affected or remote areas, while e-learning platforms ensure the continuity of workforce training despite physical disruptions. Evidence from low-resource environments suggests that these tools may provide beneficial support when appropriately implemented [50,51]. Also, similar evidence from the COVID-19 pandemic suggests that telemedicine may help maintain continuity of care during crises, provided that sufficient digital infrastructure, user training, and equitable access to technology are available. This is consistent with our findings on the importance of digital health for strengthening health system resilience during armed conflict [52]. Nonetheless, technological investment without reliable electricity, internet connectivity, and adequate digital literacy risks exacerbating inequities rather than reducing them. Consequently, digital transformation should be integrated within comprehensive emergency preparedness frameworks and surge capacity planning. Countries that had pre-established minimal response plans, incident command structures, and flexible hospital capacity were reported to have experienced comparatively better outcomes during crises [53]. Implementation of digital health may be constrained by practical barriers, especially in low- and middle-income countries. Limited financial resources, damaged infrastructure, workforce capacity limitations, and weak institutional readiness can affect the expansion of digital health, decentralized service delivery, and emergency preparedness systems. In addition, governance challenges, fragmented coordination, regulatory gaps, and broader political and economic constraints may delay implementation and limit the potential benefits of these interventions, as previously reported in Iran [54,55].

### Community engagement, adaptive training, and post-conflict recovery

At a broader level, the interplay between effective governance and community participation forms the social foundation of resilience. Public trust and collaboration with civil society organizations are considered important factors for successful health interventions in fragile settings [56,57]. Furthermore, alignment between humanitarian and development actors may reduce duplication, prevents resource fragmentation, and facilitates a smoother transition from emergency response to recovery and reconstruction [58]. Post-conflict reconstruction, therefore, should not be limited to physical rehabilitation of facilities; rather, it may incorporate principles of equity, gender responsiveness, and climate resilience, ensuring systemic redesign rather than mere restoration. Experiences from multiple post-conflict countries suggest that rebuilding efforts anchored in strengthened primary care systems and locally trained health workers may contribute to more sustainable outcomes [37,53].

### Policy implications for wartime health system resilience

Collectively, the nine policy domains identified in this study, including health workforce strengthening, digital health development, governance reform, supply chain stabilization, essential service prioritization, emergency preparedness, adaptive training systems, community engagement, and equitable post-conflict reconstruction, were frequently reported as strategies that may support service continuity and resilience in conflict settings.

### Study limitations

This study has some limitations. As a scoping review, it relied mainly on published literature, which may have introduced the possibility of publication bias. Also, except for Iranian national documents, grey literature was not systematically included; therefore, some relevant evidence may not have been captured.

## Conclusion

The findings of this research suggest that managing health systems in wartime requires a shift from reactive crisis management to a resilient and adaptive systems approach. International evidence identified in this scoping review consistently reported strategies related to strengthening governance, protecting and supporting the health workforce, maintaining essential health services, ensuring resilient supply chains, investing in digital health, enhancing emergency preparedness, sustaining medical education, promoting community engagement, and supporting equitable recovery. These priorities were largely consistent with the Iranian document review, which highlighted the importance of coordinated crisis governance, domestic pharmaceutical production, rapid activation of emergency response mechanisms, flexible service delivery, and the commitment of the health workforce during recent conflicts.

Taken together, the international evidence and the Iranian experience suggest that these policy priorities should be implemented across different phases of conflict. During the preparedness phase, activities may include strengthening emergency preparedness plans, workforce training, establishing strategic stockpiles, and investing in digital health infrastructure at both national and health system levels. During the acute phase of conflict, Ministries of Health, hospitals, emergency medical services, and primary care networks may prioritize emergency coordination, workforce protection, continuity of essential health services, and resilient supply chains. Particular attention should be given to maintaining equitable access to essential services for internally displaced persons, pregnant women, children, older adults, and individuals with chronic diseases, as these groups were consistently identified as being disproportionately affected in the international literature and were also recognized in the Iranian document review as requiring targeted support during crises. These are consistent with both international experiences and the Iranian response, where coordinated governance and pharmaceutical management supported continuity of care. During the recovery phase, governments and health organizations may focus on rebuilding health infrastructure, restoring medical education, strengthening governance, supporting workforce retention and well-being, and incorporating lessons learned into future preparedness planning.

## Supplementary Material

Additional file 1.docx

Additional file 3.docx

Checklist.pdf

Additional file 2.docx

## Data Availability

All data supporting the findings of this study are available within the paper and its Supplementary Information.

## References

[1] Shen G, Martelli P, Knox-Clarke P, et al. Health care in times of war. AMP. 2020;36:744–14. doi: 10.5465/amp.2019.0037

[2] Truppa C, Yaacoub S, Valente M, et al. Health systems resilience in fragile and conflict-affected settings: a systematic scoping review. Confl Health. 2024;18:1–18. doi: 10.1186/s13031-023-00560-7 38172918 PMC10763433

[3] Jamal Z, Alameddine M, Diaconu K, et al. Health system resilience in the face of crisis: analysing the challenges, strategies and capacities for UNRWA in Syria. Health Policy Plan. 2020;35:26–35. doi: 10.1093/heapol/czz129 31625558

[4] Campbell J, Cometto G, Rasanathan K, et al. Improving the resilience and workforce of health systems for women’s, children’s, and adolescents’ health. BMJ. 2015;351:32–35. doi: 10.1136/BMJ.h4148 26371216

[5] Chol C, Negin J, Garcia-Basteiro A, Gebrehiwot T, Debru B, Chimpolo M, et al. Health system reforms in five sub-Saharan African countries that experienced major armed conflicts (wars) during 1990–2015: a literature review. Glob Health Action. 2018;11:1–15. doi: 10.1080/16549716.2018.1517931 30270772 PMC7011843

[6] Dale E, Novak J, Dmytriiev D, et al. Resilience of primary health care in Ukraine: challenges of the pandemic and war. Health Syst Reform [Internet]. 2024;10:1–8. doi: 10.1080/23288604.2024.2352885 38875441

[7] Duclos D, Ekzayez A, Ghaddar F. Localisation and cross-border assistance to deliver humanitarian health services in North-West Syria: a qualitative inquiry for the Lancet-AUB commission on Syria. Confl Health. 2019;13:1–10. doi: 10.1186/s13031-019-0207-z 31149026 PMC6537164

[8] Ladadwa R, Hariri M, Alatras M, et al. Health information management systems and practices in conflict-affected settings: the case of northwest Syria. Global Health. 2024;20:1–19. doi: 10.1186/s12992-024-01052-w 38845021 PMC11155176

[9] Aromataris E, Munn Z. JBI reviewer’s manual PDF. JBI. 2020. Available from: https://jbi-global-wiki.refined.site/space/MANUAL/355862497/10.+Scoping+reviews

[10] Tricco AC, Lillie E, Zarin W, et al. PRISMA extension for scoping reviews (PRISMA-ScR): checklist and explanation. Ann Intern Med. 2018;169:467–473. doi: 10.7326/M18-0850 30178033

[11] Hsieh HF, Shannon SE. Three approaches to qualitative content analysis. Qual Health Res. 2005;15:1277–1288. doi: 10.1177/1049732305276687 16204405

[12] Ahsan S. Ukrainian health workers respond to war. Lancet. 2022;399:896. doi: 10.1016/S0140-6736(22)00413-5 35248177

[13] Aleksandrenko H, Shevchenko M, Chervak O. Digital health intervention reconnects war-affected people living with HIV to healthcare: Ukraine case study. Oxford Open Digit Health. 2025;3:1–12. doi: 10.1093/oodh/oqaf001 PMC11998593 40235841

[14] Dinca DV, Nicolescu CE, Dumitrica CD, et al. The impact of the Ukrainian war on the resilience and sustainability of the local public administration in Romania: an exploratory study. Rom J Eur Aff. 2023;23:64–83.

[15] Lazarus L, McClarty LM, Herpai N, et al. “…because the social work never ends”: a qualitative study exploring how NGOs responded to emerging needs while upholding responsibility to HIV prevention and treatment during the war in Ukraine. J Int AIDS Soc. 2024;27:13–20. doi: 10.1002/jia2.26309 PMC11258434 39030857

[16] WHO. Three years of war: rising demand for mental health support, trauma care and rehabilitation WHO2025. Available from: https://www.who.int/europe/news/item/24-02-2025-three-years-of-war-rising-demand-for-mental-health-support-trauma-care-and-rehabilitation

[17] Alsoukhni MA, Aziz MIA, Qosa H, et al. Public health in Sudan: priorities, challenges, and pathways to resilience in crisis. Front Public Health. 2025;13:1–7. doi: 10.3389/fpubh.2025.1647917 PMC12378227 40873986

[18] Hussein Ahmed AA, Ali Eltahir SE, Ahmed Alabass SO, et al. The ravages of war: challenges and resilience in medical education in Sudan. Educ Health. 2024;37:270–276. doi: 10.62694/efh.2024.154

[19] Mahgoub EAA, Osman S, Haga MB, et al. Dental education amid armed conflict in Sudan: unveiling the impact on training. PLOS ONE. 2024;19:1–16. doi: 10.1371/journal.pone.0311583 PMC11463757 39383150

[20] Alshawwaf A, AlNajjar O, Sultan I, et al. Resilience amid chaos: the role of Gaza medical points. PLOS Glob Public Health. 2025;5:1–12. doi: 10.1371/journal.pgph.0005073 PMC12443234 40961024

[21] Levi H, Givaty G, Ovadia YS, et al. Evaluating emergency response at a hospital near the Gaza border within 24 h of increased conflict. BMC Emerg Med. 2024;24:1–9. doi: 10.1186/s12873-024-00964-5 38515061 PMC10956292

[22] Obel J, Martin AIC, Mullahzada AW, et al. Resilience to maintain quality of intrapartum care in war torn Yemen: a retrospective pre-post study evaluating effects of changing birth volumes in a congested frontline hospital. BMC Pregnancy Childbirth. 2021;21:1–10. doi: 10.1186/s12884-020-03507-5 33413161 PMC7791801

[23] Tappis H, Elaraby S, Elnakib S, et al. Reproductive, maternal, newborn and child health service delivery during conflict in Yemen: a case study. Confl Health. 2020;14:1–16. doi: 10.1186/s13031-020-00269-x 32514295 PMC7254736

[24] Silwal RC, Jimba M, Poudyal AK, et al. Improving immunization services under the armed conflict in rural Nepal. Public Health. 2006;120:805–808. doi: 10.1016/j.puhe.2006.05.019 16879846

[25] Tang K, Zhao YX. Health system strengthening in post-conflict ethnic regions of Northeastern Myanmar: a qualitative study. Health Policy Plan. 2019;34:151–159. doi: 10.1093/heapol/czz016 30938434

[26] Tiembré I, Benié J, Coulibaly A, et al. Impact of armed conflict on the health care system of a sanitary district in Cote d’Ivoire. Med Tropicale (Mars). 2011;71:249–252. 21870550

[27] Tyndall JA, Ndiaye K, Weli C, et al. The relationship between armed conflict and reproductive, maternal, newborn and child health and nutrition status and services in northeastern Nigeria: a mixed-methods case study. Confl Health. 2020;14:1–15. doi: 10.1186/s13031-020-00318-5 33292426 PMC7664044

[28] Uwishema O. Rwanda’s health-care transformation: a case study for war-torn countries. Lancet. 2023;401:1076–1077. doi: 10.1016/S0140-6736(23)00452-X 37003693

[29] Yagos WO, Tabo Olok G, Ovuga E. Use of information and communication technology and retention of health workers in rural post-war conflict Northern Uganda: findings from a qualitative study. BMC Med Inf Decis Mak. 2017;17:1–8. doi: 10.1186/s12911-016-0403-3 PMC5223482 28068980

[30] Bayoulou S, Somé V, Niare B, et al. Health system resilience in countries facing terrorist threats: a scoping review. Discov Public Health. 2024;21:1–18. doi: 10.1186/s12982-024-00257-z

[31] Bogale B, Scambler S, Mohd Khairuddin AN, et al. Health system strengthening in fragile and conflict-affected states: a review of systematic reviews. PLOS ONE. 2024;19:1–33. doi: 10.1371/journal.pone.0305234 PMC11178226 38875266

[32] Onvlee O, Kok M, Buchan J, et al. Human resources for health in conflict affected settings: a scoping review of primary peer reviewed publications 2016–2022. Int J Health Policy Manag. 2023;12:7306–7316. doi: 10.34172/ijhpm.2023.7306 38618826 PMC10590254

[33] Singh NS, Ataullahjan A, Ndiaye K, et al. Delivering health interventions to women, children, and adolescents in conflict settings: what have we learned from ten country case studies? Lancet. 2021;397:533–542. doi: 10.1016/S0140-6736(21)00132-X 33503459

[34] WHO. Health systems recovery in fragile and conflict-affected situations in the Eastern Mediterranean Region. 2025. Available from: https://applications.emro.who.int/docs/Health-systems-recovery-eng.pdf

[35] Woodward A, Fyfe M, Handuleh J, et al. Diffusion of e-health innovations in ‘post-conflict’ settings: a qualitative study on the personal experiences of health workers. Hum Resour Health. 2014;12:1–10. doi: 10.1186/1478-4491-12-22 24754997 PMC4021507

[36] Barasa E, Mbau R, Gilson L. What is resilience and how can it be nurtured? A systematic review of empirical literature on organizational resilience. Int J Health Policy Manag. 2018;7:491–503. doi: 10.15171/ijhpm.2018.06 29935126 PMC6015506

[37] Blanchet K, Nam S, Ramalingam B, et al. Governance and capacity to manage resilience of health systems: towards a new conceptual framework. Int J Health Policy Manag. 2017;6:431–435. doi: 10.15171/ijhpm.2017.36 28812842 PMC5553211

[38] Haldane V, De Foo C, Abdalla S, et al. Health systems resilience in managing the COVID-19 pandemic: lessons from 28 countries. Nat Med. 2021;27:964–980. doi: 10.1038/s41591-021-01381-y 34002090

[39] Bertone M, Jowett M, Dale E, et al. Health financing in fragile and conflict-affected settings: what do we know, seven years on? Soc Sci Med. 2019;1:209–219. 10.1016/j.socscimed.2019.04.019 31102931

[40] Lal A, Erondu N, Heymann D, et al. Fragmented health systems in COVID-19: rectifying the misalignment between global health security and universal health coverage. Lancet. 2021;397:61–67. doi: 10.1016/S0140-6736(20)32228-5 33275906 PMC7834479

[41] Raven J, Wurie H, Witter S. Health workers’ experiences of coping with the Ebola epidemic in Sierra Leone’s health system: a qualitative study. BMC Health Serv Res. 2018;18:1–9. doi: 10.1186/s12913-018-3072-3 29622025 PMC5887191

[42] Abimbola S, Negin J, Martiniuk A, et al. Institutional analysis of health system governance. Health Policy Plan. 2017;32:1337–1344. doi: 10.1093/heapol/czx083 28981658

[43] Khodayari-Zarnaq R, Ghasemyani S, Mobasseri K, et al. Challenges for health workforce in the Iranian healthcare system: a scoping review. BMC Health Serv Res. 2025;25:1–8. doi: 10.1186/s12913-025-13167-w 41088117 PMC12522801

[44] Roberton T, Carter E, Chou V, et al. Early estimates of the indirect effects of the COVID-19 pandemic on maternal and child mortality in low-income and middle-income countries: a modelling study. The Lancet Global Health. 2020;8:e901–8. doi: 10.1016/S2214-109X(20)30229-1 32405459 PMC7217645

[45] Spiegel P, Checchi F, Colombo S, et al. Health-care needs of people affected by conflict: future trends and changing frameworks. Lancet. 2010;375:341–345. doi: 10.1016/S0140-6736(09)61873-0 20109961

[46] Mostafaei A, Khodayari-Zarnaq R, Sadeghi-Ghyassi F, et al. “Everything has been dramatically changed since the outbreak began”: a descriptive qualitative study of cancer care experiences of patients and oncology nurses during the COVID-19 pandemic. Iran J Nurs Midwifery Res. 2025;30:255–262. 40275911 10.4103/ijnmr.ijnmr_4_24 PMC12017649

[47] Yadav P. Health product supply chains in developing countries: diagnosis of the root causes of underperformance and an agenda for reform. Health Syst Reform [Internet]. 2015;1:142–154. doi: 10.4161/23288604.2014.968005 31546312

[48] Hanson K, Brikci N, Erlangga D, et al. The Lancet global health commission on financing primary health care: putting people at the centre. Lancet Global Health. 2022;10:e715–72. doi: 10.1016/S2214-109X(22)00005-5 35390342 PMC9005653

[49] Khodayari-Zarnaq R, Mobasseri K, Ghasemyani S, et al. Challenges and weaknesses of leadership and governance-related health policies in Iran: a systematic review. Arch Iran Med. 2024;27:508–521. doi: 10.34172/aim.28907 39465526 PMC11496596

[50] Rose S. Medical student education in the time of COVID-19. JAMA. 2020;323:2131–2132. doi: 10.1001/jama.2020.5227 32232420

[51] Smith A, Thomas E, Snoswell C, et al. Telehealth for global emergencies: implications for coronavirus disease 2019 (COVID-19). J Telemed Telecare. 2020;26:309–313. doi: 10.1177/1357633X20916567 32196391 PMC7140977

[52] Mostafaei A, Sadeghi-Ghyassi F, Kabiri N, et al. Experiences of patients and providers while using telemedicine in cancer care during COVID-19 pandemic: a systematic review and meta-synthesis of qualitative literature. Support Care Cancer. 2022;30:10483–10494. doi: 10.1007/s00520-022-07415-6 36322247 PMC9628519

[53] Kruk M, Ling E, Bitton A, et al. Building resilient health systems: a proposal for a resilience index. BMJ. 2017;357:1–8. doi: 10.1136/BMJ.j2323 28536191

[54] Hosseini SM, Boushehri SA, Alimohammadzadeh K. Challenges and solutions for implementing telemedicine in Iran from health policymakers’ perspective. BMC Health Serv Res. 2024;24:1–11. doi: 10.1186/s12913-023-10488-6 38200535 PMC10782789

[55] Mehrolhassani MH, Yazdi-Feyzabadi V, Dehnavieh R, et al. Barriers to telemedicine establishment in Iran: a systematic review. Iran J Public Health. 2025;54:739–750. doi: 10.18502/ijph.v54i4.18412 40321912 PMC12045879

[56] Barker K, Ling E, Fallah M, et al. Community engagement for health system resilience: evidence from Liberia’s Ebola epidemic. Health Policy Plan. 2020;35:416–423. doi: 10.1093/heapol/czz174 32040166

[57] Gilson L. Trust and the development of health care as a social institution. Soc Sci Med. 2003;56:1453–1468. doi: 10.1016/S0277-9536(02)00142-9 12614697

[58] Citaristi I. Organisation for economic co-operation and development—OECD in the Europa directory of international organizations. Routledge. 2022:694–701.

