# Supplementary material for: Managing health systems under armed conflict: a scoping review of international evidence and a document review of Iranian experiences
Source: Glob Health Action. 2026 Aug 18;19(1):2717482. doi: 10.1080/16549716.2026.2717482 (PMC13487850; doi:10.1080/16549716.2026.2717482)
Supplement: Supplementary file 2 [file ZGHA_A_2717482_SM0381.docx]

Additional file 2. Main characteristics of included studies in the scoping review phase

| **Citation** | **Country** | **Study type** | **Population / sample** | **Challenges & threats** | **Solutions & interventions** | **Policy recommendations** |
| --- | --- | --- | --- | --- | --- | --- |
| Ahsan 2022 (1) | Ukraine | Short article | Health personnel | Workforce reduction, psychological stress | Individual resilience, system flexibility | Strengthen resilience and emergency training |
| Aleksandrenko 2025 (2) | Ukraine | Case study | HIV patients | Disruption of care due to war | Establish digital health platform (ARTporuch#) to maintain contact with HIV patients during war (includes chatbot, website, and information campaign) | Invest in digital health |
| Alshawwaf 2025 (3) | Gaza | Cross-sectional | Essential health providers | Shortages of essential medicines including insulin, epilepsy drugs, cancer therapies, antibiotics, psychiatric medications (>90% of cases); lack of equipment like oxygen and diagnostic tools; shortage of trained personnel | Establish vital and mobile service points (Medical points) | Expand telemedicine coverage, remote training for providers, provide psychosocial support for frontline healthcare workers |
| Alsoukhni 2025 (4) | Sudan – armed conflict | Perspective / expert workshop analysis (EMPHNET 2024 forum) | Workshop speakers and participants + secondary data on health situation | Health system collapse, workforce shortages, service disruption, disease outbreaks, insufficient international response, lack of financial resources | Strengthen supply chains, decentralize resources to ensure access to essential medicines during crises, intersectoral collaboration, capacity building and workforce training, community-based approaches, crisis-sensitive financing | Allocate sustainable resources, decentralize services, strengthen multi-sector collaboration |
| Bayoulou 2024 (5) | Global | Scoping review | Selected studies from PubMed/Medline, CINAHL, Global Health, PsycInfo | Insufficient health system preparedness for sudden shocks; weaknesses in workforce, services, alert systems, and preparedness; absence of minimum emergency response plans | Increase capacity to absorb and adapt resources, emergency preparedness, organizational flexibility, improve stakeholder coordination, stronger health information systems | Systematically integrate preparedness, flexibility, and resilience into health policies; emergency planning and intersectoral collaboration are key |
| Bogale 2024 (6) | Global | Systematic review of reviews | 27 systematic reviews | Migration of human capital due to security concerns and harsh working conditions, limited training and resources | Community engagement, financial and support mechanisms | Adopt policies to incentivize health workforce, build local capacity, strengthen training, integrate health services, build trust, ensure monitoring and support, implement e-health solutions |
| Dale 2024 (7) | Ukraine | Short article (analysis) | Primary healthcare system analysis under COVID-19 and war pressures | Access challenges for displaced populations, shifting patient profiles and needs (esp. non-communicable and mental health), dependence on local financial resources and financial sustainability | Leverage strategic adaptations from COVID-19 to strengthen resilience during war, use digital communication and skill training, managerial and financial autonomy for rapid response | Adopt coordinated approach between humanitarian response, reconstruction, and development programs to ensure sustainability and effectiveness of PHC |
| Dincă 2023 (8) | Romania (due to Ukraine war) | Mixed methods | Local government offices affected by Ukraine war | Financial and human pressure, weak resilience, lack of prior preparedness | Develop and implement public policies to reduce crisis risks and prevent negative effects; promote continuous improvement through PDCA cycle | Adopt integrated, interdisciplinary approach engaging citizens and NGOs |
| Hussein Ahmed 2024 (9) | Sudan – armed conflict | Short analytical article (literature review) | Medical education status during armed conflict | Destruction and disruption of medical education due to conflict; disruption in educational resources, classrooms, and labs; decreased morale and wellbeing of students and faculty; shortage of teaching staff and disruption in supervision by higher education authorities | - Engagement of government, humanitarian organizations, universities, and community- Strengthening collaboration and alliance among medical schools- Use of remote learning and innovative approaches such as e-learning, PBL, TBL, small group learning, and mobile clinical training | - Establish national and international networks to support medical education- Invest in digital education platforms and develop scientific skills for crisis situations- Plan for protection of students and faculty, and rebuild infrastructure post-conflict |
| Lazarus 2024 (10) | Ukraine | Qualitative | Key NGO representatives working with vulnerable groups (HIV, LGBTQ+, drug users) | Physical and psychological impact on NGO staff, insufficient compensation | Strengthen NGOs with adequate resources | – |
| Levi 2024 (11) | Gaza | Retrospective case study | Patients presenting to emergency department within first 24 hours | High patient load after missile attacks; direct risk to staff and emergency infrastructure | – | Optimize emergency preparedness |
| Mahgoub 2024 (12) | Sudan | Mixed methods | Dental schools | Military attacks, looting, military use of buildings, disruption of clinical training | Online training, collaboration with other universities, overseas training, establishment of regional centers | Strengthen education resilience, develop regional centers, prepare for crises |
| Obel 2021 (13) | Yemen | Retrospective pre-post | Deliveries in women’s hospital in conflict zone | – | Monitor changes in service quality indicators, determine optimal staffing ratios, optimize organizational structure | – |
| Onvlee 2023 (14) | Conflict-affected countries (World Bank list) | Scoping review | Studies on health workforce management in conflict | – | – | Need for HR policies aligned with socio-political context and health workforce support at all crisis stages |
| Silwal 2006 (15) | Rural Nepal | Cross-sectional | Rural health centers for vaccination coverage | Vaccine transport and personal safety risk; low uptake due to fear of conflict | – | Use local human resources for vaccination at health centers |
| Singh 2021 (16) | 10 countries (Afghanistan, Colombia, Congo, Mali, Nigeria, Pakistan, Somalia, South Sudan, Syria, Yemen) | Multi-country case study | Reproductive, maternal, neonatal, child, and adolescent health services | Many essential maternal and child health services incomplete or not implemented; lack of comprehensive newborn mortality and adolescent care services; limited, low-quality reporting in conflict; complex security, political, and structural impacts on service delivery | Prioritize essential services: antenatal care, emergency obstetrics, child vaccination, common disease treatment, nutrition, and malnutrition management; innovative humanitarian approaches to bring services closer to populations | Ensure continuity of comprehensive RMNCAH services, empower local actors, use adaptive service delivery approaches, consider political-security context in decision-making |
| Tang 2019 (17) | Myanmar | Qualitative | Policymakers, senior managers, service providers | – | – | Health system decentralization |
| Tappis 2020 (18) | Yemen | Qualitative (case study) | Government officials, humanitarian staff, service providers | Limited access, insecurity, politicization of aid, low health system capacity, high service costs, cholera outbreak | Focus on access, quality, and service coordination; invest in HR development and supply management | Remove political barriers, ensure full service packages via investment in skilled workforce, decentralize health services, overcome cost barriers, strengthen supply chain management |
| Tiembré 2011 (19) | Côte d’Ivoire – armed conflict | Cross-sectional | Health centers and staff in Seguela region | 60.7% centers inactive, 77.7% qualified staff gone, 46.2% infrastructure damaged; reduced service access, increased measles and STIs, emergence of gastritis and trauma-related conditions | – | Return qualified health workforce, rebuild/renovate health centers to restore service access |
| Truppa 2024 (20) | Global (studies from Africa, Middle East, and other conflict-affected areas) | Scoping review | – | Lack of coherent governance, insecurity, distrust, weak data | Absorptive, adaptive, transformative operations in seven operational domains | Strengthen governance, data-centric approaches, HR support, and transformative capacity |
| Tyndall 2020 (21) | Nigeria | Mixed methods (quant + qual) | 60+ interviews with government, international organizations, NGOs; quantitative data from DHS, MICS, NNHS over selected years | Poor security, health workforce shortages, insufficient financial resources | Child vaccination, integrated childhood disease management (IMCI) focusing on malaria, pneumonia, malnutrition, diarrheal diseases; maternal, newborn, and child health services including EmONC/BEmONC; reproductive health including HIV services and GBV programs; NCD management; mental health and psychosocial support | Need for humanitarian support with commitment from federal, state, local governments, plus external financing and technical assistance |
| Uwishema 2023 (22) | Rwanda (post-1994 genocide) | Short letter | National health system post-crisis | Disease outbreaks (HIV, malaria, TB), lack of health specialists, inadequate infrastructure and resources | Emphasize community-based health, decentralize services, develop accountable workforce, invest in health tech and information systems | Proper investment and strong commitment to health, community participation |
| WHO 2025 (23) | Eastern Mediterranean countries | Guideline | Health system recovery post-war | – | – | Do no harm, respect humanitarian principles, apply development effectiveness principles, integrate reconstruction from start, align with multi-sector frameworks, engage local communities, promote gender-responsive and accountable approaches, adopt life-cycle approach, integrate climate and disaster resilience |
| WHO 2025 (24) | Ukraine | News report | Ukrainian population | >2,254 attacks on health facilities over 3 years (including pediatric clinics); sharp increase in mental health needs (46% affected); rise in neurological and psychiatric disorders (41% & 39%); pressure on primary care, limited access for displaced populations; NCDs account for 84% of deaths; shortage of trauma, prosthetics, rehab specialists | – | Capacity building, prioritize primary care and critical disease care (NCDs, vaccination, mental health, HIV, TB), infection control, combat antimicrobial resistance |
| Woodward 2014 (25) | Post-conflict settings: Liberia, West Bank & Gaza, Sierra Leone, Somaliland | Qualitative | Health worker perspectives on e-health post-conflict | Weak, unreliable, inaccessible internet | – | Optimal investment in e-health, raise staff awareness, remove physical and financial barriers to internet access |
| Yagos 2017 (26) | Uganda | Qualitative | Health worker perspectives on e-health in rural post-war areas | Challenges in implementing health IT: low confidence, knowledge, and skills; poor internet; insufficient computers; unstable electricity; lack of modems, costly access to off-site computers | – | Targeted investment in rural health IT infrastructure, train health workers |

1.	Ahsan S. Ukrainian health workers respond to war. Lancet. 2022;399(10328):896.

2.	Aleksandrenko H, Shevchenko M, Chervak O. Digital health intervention reconnects war-affected people living with HIV to healthcare: Ukraine case study. Oxf Open Digit Health. 2025;3:oqaf001.

3.	Alshawwaf A, AlNajjar O, Sultan I, Qunaibi E. Resilience amid chaos: The role of Gaza medical points. PLOS GLOBAL PUBLIC HEALTH. 2025;5(9).

4.	Alsoukhni MA, Aziz MIA, Qosa H, Bani I, Omar M, Elhassan MMO, et al. Public health in Sudan: priorities, challenges, and pathways to resilience in crisis. Front Public Health. 2025;13:1647917.

5.	Bayoulou S, Somé V, Niare B, Rouamba TD, Zoungrana W, Kollo AT, et al. Health system resilience in countries facing terrorist threats: a scoping review. DISCOVER PUBLIC HEALTH. 2024;21(1).

6.	Bogale B, Scambler S, Mohd Khairuddin AN, Gallagher JE. Health system strengthening in fragile and conflict-affected states: A review of systematic reviews. PloS one. 2024;19(6):e0305234.

7.	Dale E, Novak J, Dmytriiev D, Demeshko O, Habicht J. Resilience of Primary Health Care in Ukraine: Challenges of the Pandemic and War. Health systems and reform. 2024;10(1):2352885.

8.	Dinca DV, Nicolescu CE, Dumitrica CD, Dogaru TC. The Impact of the Ukrainian War on the Resilience and Sustainability of the Local Public Administration in Romania: An Exploratory Study. ROMANIAN JOURNAL OF EUROPEAN AFFAIRS. 2023;23(2):64-83.

9.	Hussein Ahmed AA, Ali Eltahir SE, Ahmed Alabass SO, Mohamed Osman FH. The ravages of war: challenges and resilience in medical education in Sudan. Education for Health. 2024;37(3):270-6.

10.	Lazarus L, McClarty LM, Herpai N, Pavlova D, Tarasova T, Gnatenko A, et al. "…because the social work never ends": a qualitative study exploring how NGOs responded to emerging needs while upholding responsibility to HIV prevention and treatment during the war in Ukraine. J Int AIDS Soc. 2024;27 Suppl 3(Suppl 3):e26309.

11.	Levi H, Givaty G, Ovadia YS, Alon Y, Saban M. Evaluating emergency response at a hospital near the Gaza border within 24 h of increased conflict. BMC Emerg Med. 2024;24(1):47.

12.	Mahgoub EAA, Osman S, Haga MB, Khairy A, Osman SHM, Hassan AMA, et al. Dental education amid armed conflict in Sudan: Unveiling the impact on training. PloS one. 2024;19(10).

13.	Obel J, Martin AIC, Mullahzada AW, Kremer R, Maaløe N. Resilience to maintain quality of intrapartum care in war torn Yemen: a retrospective pre-post study evaluating effects of changing birth volumes in a congested frontline hospital. BMC Pregnancy Childbirth. 2021;21(1):36.

14.	Onvlee O, Kok M, Buchan J, Dieleman M, Hamza M, Herbst C. Human Resources for Health in Conflict Affected Settings: A Scoping Review of Primary Peer Reviewed Publications 2016–2022. International Journal of Health Policy and Management. 2023;12(1).

15.	Silwal RC, Jimba M, Poudyal AK, Poudel KC, Wakai S. Improving immunization services under the armed conflict in rural Nepal. PUBLIC HEALTH. 2006;120(9):805-8.

16.	Singh NS, Ataullahjan A, Ndiaye K, Das JK, Wise PH, Altare C, et al. Delivering health interventions to women, children, and adolescents in conflict settings: what have we learned from ten country case studies? The Lancet. 2021;397(10273):533-42.

17.	Tang K, Zhao YX. Health system strengthening in post-conflict ethnic regions of Northeastern Myanmar: a qualitative study. HEALTH POLICY AND PLANNING. 2019;34(2):151-9.

18.	Tappis H, Elaraby S, Elnakib S, AlShawafi NAA, BaSaleem H, Al-Gawfi IAS, et al. Reproductive, maternal, newborn and child health service delivery during conflict in Yemen: a case study. Confl Health. 2020;14:30.

19.	Tiembré I, Benié J, Coulibaly A, Dagnan S, Ekra D, Coulibaly S, et al. [Impact of armed conflict on the health care system of a sanitary district in Cote d'Ivoire]. Med Trop (Mars). 2011;71(3):249-52.

20.	Truppa C, Yaacoub S, Valente M, Celentano G, Ragazzoni L, Saulnier D. Health systems resilience in fragile and conflict-affected settings: a systematic scoping review. Conflict and Health. 2024;18(1).

21.	Tyndall JA, Ndiaye K, Weli C, Dejene E, Ume N, Inyang V, et al. The relationship between armed conflict and reproductive, maternal, newborn and child health and nutrition status and services in northeastern Nigeria: a mixed-methods case study. Confl Health. 2020;14(1):75.

22.	Uwishema O. Rwanda's health-care transformation: a case study for war-torn countries. The Lancet. 2023;401(10382):1076-7.

23.	WHO. Health systems recovery in fragile and conflict-affected situations in the Eastern Mediterranean Region. 2025.

24.	WHO. Three years of war: rising demand for mental health support, trauma care and rehabilitation. 2025.

25.	Woodward A, Fyfe M, Handuleh J, Patel P, Godman B, Leather A, et al. Diffusion of e-health innovations in 'post-conflict' settings: A qualitative study on the personal experiences of health workers. Human Resources for Health. 2014;12(1).

26.	Yagos WO, Tabo Olok G, Ovuga E. Use of information and communication technology and retention of health workers in rural post-war conflict Northern Uganda: findings from a qualitative study. BMC Med Inform Decis Mak. 2017;17(1):6.
