# Supplementary material for: Managing health systems under armed conflict: a scoping review of international evidence and a document review of Iranian experiences
Source: Glob Health Action. 2026 Aug 18;19(1):2717482. doi: 10.1080/16549716.2026.2717482 (PMC13487850; doi:10.1080/16549716.2026.2717482)
Supplement: Supplementary file 3 [file ZGHA_A_2717482_SM0383.docx]

Additional file 3. Main characteristics of included national documents in the document review phase

| **Author / Interviewee organization** | **Related war or crisis** | **Health system challenges and weaknesses** | **Health system strengths** | **Solutions and recommendations for improvement** |
| --- | --- | --- | --- | --- |
| Shahid Beheshti University of Medical Sciences | 12-Day Imposed War (Bombings and incidents such as Qods Square, Evin Prison, IRIB) | Sudden and unexpected onset of war; high volume of casualties in a short time; extreme risks for healthcare staff (structural/systemic weaknesses not explicitly mentioned) | Full and continuous presence of healthcare staff at all levels; uninterrupted delivery of healthcare services; high commitment beyond shifts; rapid and precise surgeries under supervision of professors; high hospital response capacity (24% of war casualties nationally, ~35% of all university casualties); rapid management of casualties without pre-hospital emergency in some incidents; deployment of doctors from other university centers to strengthen response | Not specified |
| Ministry of Health | 12-Day War (Summer 1404) alongside economic pressures, sanctions, and security consequences | Simultaneous pressures of war, sanctions, and economic issues; high public demand for healthcare; shortage of human resources; limited in-person monitoring during crisis; weak infrastructure; shortage of some equipment; communication problems during war | Rapid dissemination of war protocols to medical centers; ensured 100% emergency readiness; implementation of mass casualty management guidelines (MCM); extensive training with webinars attended by over 2,800 people; distribution of vital equipment (21,000+ dialysis filters to host provinces); development of crisis management indicators and checklists; no shortage of drugs/equipment per minister; care for ~5,700 injured; effective coordination among medical universities; rapid emergency organization (2–3 hours); readiness for modern warfare with management and media dimensions | Emphasis on reforming healthcare structures; strengthening infrastructure; addressing human resource shortages; improving equipment and communication infrastructure; continued training and preparedness for modern warfare (mostly implicit policy recommendations) |
| Ministry of Health | 12-Day Iran-Israel War | - Potential drug and equipment shortages in prolonged crises (despite successful management of this short war) - Need for continuous preparedness for future crises | - Care for ~5,700 injured without shortage of drugs/equipment - Operational experience and capability of healthcare staff - Continuous organization and coordination of Ministry of Health in crisis management | - Strengthening public health literacy for better crisis response - Conducting preparedness drills and experiential planning for potential crises - Continuous review and updating of protocols and logistics (drugs, blood, equipment, ambulances) |
| Ministry of Health | 12-Day War | Logistics challenges, managing high volume of casualties, direct risks to healthcare staff | Preventive logistical readiness, real-world drill exercises, prioritization of services for the public, culture of sacrifice and courage, holistic view of health | Preventive planning and strategic reserves, real drills and training, maintaining essential services, institutionalizing culture of courage and sacrifice, addressing economic and social health determinants |
| Deputy Ministry of Health and Head of Food & Drug Organization | 12-Day War (Sudden crisis and emergency) | Sudden crisis; temporary disruption in some production/distribution lines (no actual shortages reported) | Immediate establishment of Food & Drug crisis headquarters; rapid coordination with Ministry of Health, Customs, Central Bank, and distribution companies; continuous communication with health managers from day one; emergency meetings with producers and distributors; no shortages of drugs, medical supplies, or infant formula; quick restoration of production/distribution; uninterrupted pharmacy services; high domestic production capacity (380 supplement companies, 29 natural/traditional medicine companies); cohesive production-distribution-service chain; successful national crisis management | Delegation of some authorities to universities and professional associations; emphasis on strengthening infrastructure and future preparedness relying on national resilience model (general, non-detailed recommendations) |
| Parliamentary Health Commission | 12-Day War | No specific challenges mentioned; general emphasis on participation of health workforce in preparedness and response | Voluntary, committed presence of doctors, nurses, and specialists; 100% staff readiness; continuous field visits by Minister and Deputy; adequate blood reserves; overall coordination | Encourage public participation in blood donation; continue field monitoring and needs assessment of healthcare centers (general recommendations) |
| Iranian Medical Council | 12-Day War | - Lack of unified command structure in crisis management - No clear plan for keeping clinics and health centers operational during crisis - Lack of standardized public education for crises (emergency kits, evacuation routes, etc.) - Insufficient use of professional associations and disaster health graduates - Some shortcomings in volunteer organization and media management - Reduced outpatient visits during war (natural for non-essential services) | - High dedication, selflessness, and responsibility of healthcare staff; volunteer presence even in off-shifts - Scientific and practical experience of medical community based on prior wars and COVID-19 - Active presence of Health Ministry leadership - Strong human resources structure in health - Thousands of volunteer doctors available in capital and provinces - Private doctors maintained essential services | - Integrate crisis and civil defense management under presidential leadership - Develop and institutionalize public preparedness standards (emergency kits, evacuation, training) - Scientific and structured use of volunteers and disaster health graduates - Strengthen human resources and facilities for large-scale crises - Stabilize health budget and GDP allocation for health - Respect and support medical community, prevent humiliation in media, and monitor mental health personnel - Recognize and honor medical community efforts - Sustain medical community status in non-crisis periods - Invest in maintaining motivation of medical workforce as a national asset |
| Physician and Professional Activist | 12-Day Iran-Israel War | - Nature of the war different from traditional wars; entire country became operational zone - No need for traditional jihadist groups due to continued services | - No shortages in medical services or patient care - Routine medical services continued uninterrupted in public and private sectors - Medical workforce experience from prior wars and COVID-19 ensured resilience - Proper prioritization of surgical and trauma centers | - Maintain morale and motivation of medical staff, acknowledge sacrifice - Prioritize critical services over non-essential during war |
| Pharmaceutical Expert | 12-Day Iran-Israel War | - Sudden war without prior preparedness - Some specific drug shortages (e.g., chemotherapy) - Issues with raw material supply and price disputes among companies and organizations | - No serious shortages of essential drugs and supplies - Cooperation among public, private sector, Ministry of Health, Food & Drug Organization, pharmaceutical companies, and pharmacies maintained supply chain - Pharmacies managed scarce drugs to improve access - Short-term readiness and flexibility of national pharmaceutical system | - Operational planning and preparedness for potential prolonged crises - Translate paper plans into practical actions - Strengthen collaboration among public, private sector, and pharmaceutical system for future crises |
| Iranian Medical Council | 12-Day War | - Direct attacks on medical centers and clinics - Violation of international law and Geneva Conventions - Physical and mental fatigue of healthcare staff - Heavy workload and resource shortages - Damage to healthcare infrastructure and direct threats to staff - Internet outages and communication limits | - Continuous effort to document and report violations to international organizations - Uninterrupted, dedicated presence of healthcare staff even away from families - Formation of specialized committees coordinated with universities - Real solidarity among doctors, nurses, students, and emergency teams - Complete coordination among agencies for drugs and equipment | - Pursue international law and condemn inhumane actions - Document attacks and report globally - Rebuild health system infrastructure after crisis - Special support for healthcare staff and strengthen backing - Shift official mindset toward medical community - Enhance physical and mental resilience of healthcare staff during crises |
| Deputy Ministry of Health | 12-Day Iran-Israel War | - Damage to 7 hospitals (e.g., Farabi Kermanshah, Shahid Motahari, Tehran Children) - Half of hospitals fatigued, one-third needing reconstruction - No safe or underground hospitals for direct attacks - Strain on hospital beds, need for emergency hospital management - Limited disruptions in drug and blood supply in early days - Transportation and lodging challenges for healthcare staff | - Managed 5,750+ casualties and 1,100 deaths with free care - Performed 1,064 surgeries over 12 days; continued non-essential surgeries in some centers - Mobilization and coordination of medical community, volunteers, retirees, and students - Cooperation with armed forces to use military hospital capacity - Rapid drug and equipment replacement and supply | - Establish safe, resilient hospitals with defined crisis capacity - Design convalescent spaces using schools/universities if crisis prolongs - Separate hospitals for war casualties and general services - Strengthen inter-agency coordination and civil defense - Reconstruct and secure hospitals and infrastructure - Continue psychosocial support for staff and patients - Plan strategic reserves of drugs and medical equipment for prolonged crises |
| Second Vice President of Nursing Council | 12-Day Iran-Israel War | - Damage to several hospitals and evacuation of some centers - Severe psychological stress on nurses and staff - Need for chemical and radiation emergency preparedness - Shortage of sustainable human resources | - Extensive readiness of nurses, students, and retirees - Organization of volunteers within 48 hours - Continuous care for over 5,000 injured | - Strengthen human resources in health system, especially nurses and pre-hospital staff - Special attention to staff welfare and operational capacity - Public education and preparedness for emergencies |
| Emergency Department, Imam Hospital Complex | 12-Day Iran-Israel War | - Lack of secure shelters and protected hospital areas - Risk of crowding and interference with emergency response | - Crisis management experience, trained emergency, Red Crescent, and fire teams - Command and order at incident site | - Build secure shelters in hospitals and cities - Public education and emergency kit preparation (documents, water, food, whistle) - Inform public to avoid incident sites to facilitate response |
| Emergency Supervisor, Imam Hospital | 12-Day Iran-Israel War | - Staff concern for family and stress - Long and round-the-clock presence under stressful conditions | - Commitment and conscientiousness of staff; active presence even in personal hardship - Mutual support within team and crisis management | - Remind importance of public support for healthcare staff - Preserve memory and value of staff sacrifices - Pre-crisis preparedness to reduce stress and pressure |
| Civil Defense Committee, Ministry of Health | 12-Day Iran-Israel War | - High complexity with simultaneous attacks at multiple locations - Need for hospitals prepared for potential water, electricity, gas cuts - Population increase in key provinces and host cities | - Exemplary coordination among agencies and healthcare staff - Full readiness of emergency and healthcare teams - Effective management of access and service provision even with population displacement | - Strengthen critical hospital infrastructure and create backup systems - Continue preparedness programs, training, and inter-sectoral coordination - Reduce public anxiety and increase sense of security |
| Supreme Nursing Council | 12-Day War (Recent Imposed War) | - Chronic shortage of nurses - Severe workload and fatigue during crisis - Attacks on healthcare centers and ambulances - Ignored nurse demands post-crisis (e.g., COVID-19) - Unfair payment systems and delayed compensation | - Continuous hospital service despite direct attacks - High dedication of nurses, similar to Iran-Iraq War - Over 10 staff died in duty - Maintaining civil defense health during war | - Immediate recruitment and retention of nurses - Accelerate hiring exams for temporary and COVID-era nurses - Establish special nursing task force for systemic issues - Reform pay system and equitable resource distribution - Special compensations, remove progressive tax, pay dues, overtime - Implement motivational programs to increase resilience |
| Parliamentary Research Center | 12-Day Iran-Israel War | - Structural weakness in some organizations, lack of unified management - Over-focus on preliminary stages, slow operations - Dispersed resources and social networks - Fear of political consequences and self-censorship in media - Lack of legal mechanisms for public participation | - Broad and organized mobilization of people and civil organizations - Provision of essential needs (children, elderly, food, medical) - Neighborhood protection groups and volunteer services - Educational content for social anxiety control - Social resilience and crisis documentation - Cross-faction solidarity and leveraging international support | - Shift governance approach from “head/employer” to “director” guiding, supporting, and supervising - Establish cooperation mechanisms among social organizations - Provide advisory roles and facilitate NGO participation in executive/legal decisions - Empower NGOs via training, organization, and capacity building - Develop pre-designed networks for future crisis management - Institutionalize inclusive participation and mutual trust between government and people |
| Iran University of Medical Sciences | 12-Day Iran-Israel War | Direct attack on three hospitals; need to evacuate some departments; staff security threats; psychological pressure; potential human resource shortage | Rapid and coordinated hospital response; admission of 705 injured without interruption; crisis committees; trauma teams with specialists; 21 hospitals on standby; drug and dry food depots; emergency power; use of students and volunteers; mental health packages | Crisis committee formation and regular meetings; medical center readiness; prioritization of surgeries; virtual mental health assessment; design and distribution of mental health packages; rapid admission and discharge of casualties; use of student capacity; continued drug and essential supply stockpiles |
| Shahid Beheshti University of Medical Sciences | Iran-Iraq War (Defensive War) | Direct threats to healthcare staff at frontlines; managing high number of casualties; resource limitations | Field hospitals and mobile units; frontline medical teams; treatment of 8,000+ casualties in 53 field hospitals; 16–18k doctors and 60k healthcare workers; prevention of epidemics and communicable diseases | Document military health services and healthcare staff heroism; record and analyze war healthcare lessons for future generations; educational modeling for military health training |
| Iran Health Insurance Organization | 12-Day Israel-Iran War (1404) | Extra pressure on financial network; rapid response to high patient volume; ensure insurance coverage for vulnerable groups; risk of disruption if part of system shuts down | Provided over 3 million outpatient services; uninterrupted inpatient coverage; utilized 29,000 active treatment and diagnostic centers; 24-hour response system; rapid financial resource distribution; produced educational and informational content; digital coordination | Use non-face-to-face and digital platforms for services and communication; field monitoring and continuous supervision of sensitive centers; automatic extension of insurance coverage for vulnerable groups; strengthen system resilience and rapid crisis management |
| Deputy Minister of Health | 12-Day Israel-Iran War | Continuous casualty admission; hospital readiness for high patient load; weak infrastructure and emergency equipment; risk of service disruption under high pressure | Immediate MOH protocols; incident command system; allocation of backup centers and emergency capacity; coordination with relief agencies and insurers; free care for casualties | Activate crisis command systems; prioritize injured patients; manage beds and operating rooms; establish secondary stations; utilize all human and equipment capacity; focus on infrastructure preparedness and supply stockpiles |
| Physician | 12-Day Israel War | Weak health system preparedness; hospital structural vulnerability; lack of comprehensive safe centers in major cities | Commitment and dedication of healthcare staff; potential human and technical capacity | Establish specialized crisis committee; develop protocols and software standards; reinforce and secure hospitals; allocate sustainable budgets; equip operating rooms and specialized units; convert potential human capacity into active workforce |
| Minister & Head of Welfare Organization | 12-Day Israel War | Need for precise documentation; lack of participatory management from centers to headquarters; need for intersectoral coordination and quick replacement of centers | Successful management of 42 centers in Tehran Welfare Directorate; potential human capacity; intersectoral coordination; active public participation | Proper documentation and experience recording; design participatory management system from grassroots to HQ; regional zoning for center-province coordination; use 50/50 neighborhood plan for comprehensive, centralized service delivery; continuous learning and review of crisis response |
| Ministry of Health | 12-Day Israel-Iran War (1404) | Budget constraints and deductions; lack of volunteer organization; low health literacy during crisis; psychological pressure on staff | Crisis management in 12-Day War; support for nursing community; prior successful organizational experience from Iran-Iraq War; commitment to nursing tariff implementation | Organize volunteer nurses and physicians; revive emergency teams model from Iran-Iraq War; improve public health literacy; produce specialized educational content; follow up on nursing service tariffs |
| Nursing Organization | 12-Day Israel-Iran War (1404) | Severe psychological pressure on nurses (especially women); human resource resilience challenges; uneven distribution of nurses; severe shortage in some areas | Crisis headquarters formation; field visits; welcoming ~1,000 volunteers; educational content on stress management and resilience; family accommodation measures | Focus on psychological resilience of nurses; improve human resource distribution; enhance livelihood and organizational support; continue psychological and skill training during crisis |
| Analytical Report (No specific interviewee) | War and armed crises | Blocked access routes; shortage of drugs and food; logistical issues transporting elderly; lack of specialists in remote areas; psychological impact of explosions and stress; increased physical and social vulnerability of elderly | Formation of local volunteer networks; collaboration with healthcare centers for medication and nutrition; local nurse training; family and community support capacity | Create “emergency kits” for elderly; local training for families and volunteers in first aid; use simple technologies (phone, local radio) for alerts and communication; develop local support networks; provide special vehicles for elderly transport; cooperate with international organizations |
| Medical Council | Iran-Iraq War (Defensive War) – Operations such as Ramadan, Valfajr, Karbala 4 & 5, Mersad | Mismatch of resources to high casualty volume (esp. Karbala 4); shortage of personnel at peak casualties; insufficient experience with chemical attacks early in war; no written documentation of wartime medicine; long distance from frontlines early in war | Formation of voluntary university emergency teams; jihadist presence of doctors and residents; field hospitals near frontlines; reduced casualty transfer time to 15–20 minutes late in war; successful triage and trauma management; spirit of medical sacrifice | Documentation and recording of wartime medicine and Iran-Iraq War experience; preserve and strengthen volunteer teams; bring treatment lines closer to crisis; practical training in trauma and triage under mass casualty conditions |
