# Supplementary material for: Managing health systems under armed conflict: a scoping review of international evidence and a document review of Iranian experiences
Source: Glob Health Action. 2026 Aug 18;19(1):2717482. doi: 10.1080/16549716.2026.2717482 (PMC13487850; doi:10.1080/16549716.2026.2717482)
Supplement: Supplementary file 4 [file ZGHA_A_2717482_SM0384.docx]

Additional file 1. Search strategy

Search strategy for Scopus

TITLE-ABS-KEY("health system resilience" OR "health system management" OR "human resource*"OR Staffing* OR "financial management"OR "Financial Activit*" OR Workforce* OR Workforce OR "Financial Management" OR "resource generation" OR stewardship OR "healthcare provision") AND TITLE-ABS-KEY("Armed Conflicts" OR "Armed Conflict*" OR "violence" OR War OR Wars OR Atrocities OR conflict OR "terrorist threats" OR wartime)

Search strategy for ISI Web of Science

TS=("health system resilience" OR "health system management" OR "human resource*"OR Staffing* OR "financial management"OR "Financial Activit*" OR Workforce* OR Workforce OR "Financial Management" OR "resource generation" OR stewardship OR "healthcare provision") AND TS=("Armed Conflicts" OR "Armed Conflict*" OR "violence" OR War OR Wars OR Atrocities OR conflict OR "terrorist threats" OR wartime)

Search strategy for Embase

('armed conflict'/exp OR 'armed conflict*':ti,ab,kw OR war:ti,ab,kw OR wars:ti,ab,kw OR violence:ti,ab,kw OR conflict:ti,ab,kw OR atrocities:ti,ab,kw OR 'terrorist threat*':ti,ab,kw OR wartime:ti,ab,kw) AND ('health system resilience':ti,ab,kw OR 'health system management':ti,ab,kw OR 'human resource*':ti,ab,kw OR staffing:ti,ab,kw OR workforce/exp OR workforce*:ti,ab,kw OR 'financial management'/exp OR 'financial management':ti,ab,kw OR 'financial activit*':ti,ab,kw OR 'resource generation':ti,ab,kw OR stewardship:ti,ab,kw OR 'healthcare provision':ti,ab,kw)
